# Associations between HIV stigma and health-related quality-of-life among people living with HIV: cross-sectional analysis of data from HPTN 071 (PopART)

**DOI:** 10.1038/s41598-024-63216-3

**Published:** 2024-06-04

**Authors:** Emily Hall, Katherine Davis, Julius Ohrnberger, Michael Pickles, Simon Gregson, Ranjeeta Thomas, James R. Hargreaves, Triantafyllos Pliakas, Justin Bwalya, Rory Dunbar, Tila Mainga, Kwame Shanaube, Graeme Hoddinott, Virginia Bond, Peter Bock, Helen Ayles, Anne L. Stangl, Deborah Donnell, Richard Hayes, Sarah Fidler, Katharina Hauck, The HPTN 071 (PopART) study team, James R. Hargreaves, Deborah Watson-Jones, Peter Godfrey-Faussett, Kalpana Sabapathy, Katharina Hauck, Peter C. Smith, Anne Cori, Michael Pickles, Nomtha Bell-Mandla, Blia Yang, Anelet James, Redwaan Vermaak, Nozizwe Makola, Graeme Hoddinott, Vikesh Naidoo, Virginia Bond, Musonda Simwinga, Alwyn Mwinga, Barry Kosloff, Mohammed Limbada, Justin Bwalya, Chepela Ngulube, Christophe Fraser, Susan Eshleman, Yaw Agyei, Vanessa Cummings, Denni Catalano, Estelle Piwowar-Manning, Deborah Donnell, Lynda Emel, Lisa Bunts, Heather Noble, David Burns, Alain Kouda, Niru Sista, Ayana Moore, Rhonda White, Tanette Headen, Eric Miller, Kathy Hinson, Sten Vermund, Mark Barnes, Lyn Horn, Albert Mwango, Megan Baldwin, Shauna Wolf, Erin Hughes, Wafaa el-Sadr

**Affiliations:** 1 MRC Centre for Global Infectious Disease Analysis, Imperial College London, London, UK; 2 Abdul Latif Jameel Institute for Disease and Emergency Analytics, School of Public Health, Imperial College London, London, UK; 3 Department of Health Policy, London School of Economics, London, UK; 4 Department of Public Health, Environments and Society, Faculty of Public Health and Policy, London School of Hygiene and Tropical Medicine, London, UK; 5 Zambart, School of Medicine, University of Zambia, Lusaka, Zambia; 6 Desmond Tutu TB Centre, Department of Paediatrics and Child Health, Faculty of Medicine and Health, University of Stellenbosch, Cape Town, South Africa; 7 Department of Global Health and Development, Faculty of Public Health and Policy, London School of Hygiene and Tropical Medicine, London, UK; 8 Department of Clinical Research, Faculty of Infectious and Tropical Diseases, London School of Hygiene and Tropical Medicine, London, UK; 9 Hera Solutions, Baltimore, MD USA; 10 Department of International Health, Johns Hopkins University Bloomberg School of Public Health, Baltimore, MD USA; 11 Vaccine and Infectious Disease Division, Fred Hutchinson Cancer Research Center, Seattle, WA USA; 12 Department of Infectious Disease Epidemiology, Faculty of Epidemiology and Population Health, London School of Hygiene and Tropical Medicine, London, UK; 13 Department of Infectious Disease, Faculty of Medicine, Imperial College London, London, UK; 14 London School of Hygiene & Tropical Medicine, London, UK; 15 Imperial College London, London, UK; 16 Desmond Tutu TB Centre, Stellenbosch University, Stellenbosch, South Africa; 17 Zambart, University of Zambia School of Medicine, Lusaka, Zambia; 18 Nuffield Department of Medicine, Oxford University, Oxford, UK; 19 Department of Pathology, Johns Hopkins University School of Medicine, Baltimore, MD USA; 20 HIV Prevention Trials Network Statistical and Data Management Center, Statistical Center for HIV/AIDS Research and Prevention, Seattle, WA USA; 21 Division of AIDS, National Institute of Allergy and Infectious Diseases, National Institutes of Health, Bethesda, MD USA; 22 FHI 360, Durham, NC USA; 23 Yale University, New Haven, CT USA; 24 Ropes & Gray, Boston, MA USA; 25 Mailman School of Public Health, Columbia University, New York, NY USA

**Keywords:** HIV infections, Epidemiology, Public health, Quality of life

## Abstract

People living with HIV (PLHIV) report lower health-related quality-of-life (HRQoL) than HIV-negative people. HIV stigma may contribute to this. We explored the association between HIV stigma and HRQoL among PLHIV. We used cross-sectional data from 3991 randomly selected PLHIV who were surveyed in 2017–2018 for HPTN 071 (PopART), a cluster randomised trial in Zambia and South Africa. Participants were 18–44 years, had laboratory-confirmed HIV infection, and knew their status. HRQoL was measured using the EuroQol-5-dimensions-5-levels (EQ-5D-5L) questionnaire. Stigma outcomes included: internalised stigma, stigma experienced in the community, and stigma experienced in healthcare settings. Associations were examined using logistic regression. Participants who had experienced community stigma (n = 693/3991) had higher odds of reporting problems in at least one HRQoL domain, compared to those who had not (adjusted odds ratio, aOR: 1.51, 95% confidence interval, 95% Cl: 1.16–1.98, *p* = 0.002). Having experienced internalised stigma was also associated with reporting problems in at least one HRQoL domain (n = 552/3991, aOR: 1.98, 95% CI: 1.54–2.54, p < 0.001). However, having experienced stigma in a healthcare setting was less common (n = 158/3991) and not associated with HRQoL (aOR: 1.04, 95% CI: 0.68–1.58, *p* = 0.850). A stronger focus on interventions for internalised stigma and stigma experienced in the community is required.

## Introduction

Life expectancy for people living with human immunodeficiency virus (HIV) has improved substantially^1^. However, people living with HIV still have higher rates of comorbidities, and lower health-related quality-of-life than the general population^2^. Health-related quality-of-life measures a person’s wellbeing across different domains that are directly affected by their health, such as mobility and pain^3^. Concerns about health-related quality-of-life among people living with HIV led to recent suggestions that health-related quality-of-life should be an additional fourth pillar in the Joint United Nations Programme on HIV/AIDS (UNAIDS)’s 90–90-90 targets for HIV testing, treatment, and viral suppression^2^. To improve the health-related quality-of-life of people living with HIV, an understanding of the factors that influence health-related quality-of-life is needed.

It has been proposed that experiencing HIV stigma might make people living with HIV who know their status more likely to experience lower health-related quality-of-life^4,5^. HIV stigma refers to the negative behaviours and attitudes directed towards people living with HIV^6^. This stigma remains prevalent in sub-Saharan Africa, with a South African study reporting that 29% of people living with HIV surveyed had experienced discrimination or exclusion because of their status^7^. HIV stigma has been linked to adverse health outcomes including delayed access to healthcare, and reduced treatment adherence, which may lower health-related quality-of-life^8–14^. Ongoing work to reduce stigma offers an opportunity to improve health-related quality-of-life among people living with HIV in Africa, yet to do so effectively we need to understand the mechanisms by which HIV stigma might affect health-related quality-of-life^15^.

Gaining a comprehensive understanding of the relationship between HIV stigma and health-related quality-of-life in sub-Saharan African settings is challenging, however, as few relevant studies have been conducted in the region^4,5^. Furthermore, the limited existing research has often focused exclusively on individuals entering clinical care or on treatment, which does not provide a complete insight into the relationship between stigma and health-related quality-of-life among all people living with HIV in the community^4,5,8,16,17^. Use of HIV-specific measures of health-related quality-of-life is also common and makes it hard to compare the effect of stigma on wellbeing among people living with HIV against the effect of other threats to wellbeing among different population groups^4,5,8,16,17^. Finally, studies have frequently been small in scale, reducing the precision of the findings^4,5,8,16,17^. As a result, there is a lack of robust information on the relationship between HIV stigma and health-related quality-of-life, which constrains efforts to improve the health-related quality-of-life of people living with HIV.

This information is also important for economic appraisals of the benefits of interventions, which often rely on health-related quality-of-life measures^18^. The lack of clarity about the relationship between stigma and health-related quality-of-life may undermine these assessments, complicating economic evaluations of stigma reduction interventions^19,20^.

We analysed data from approximately 4,000 people living with HIV who knew their HIV status and participated in the HIV Prevention Trials Network (HPTN) 071 (PopART) trial, to assess the association between HIV stigma and health-related quality-of-life. We aimed to compare health-related quality-of-life profiles between people living with HIV who had and had not experienced HIV stigma; explore associations between three types of HIV stigma and health-related quality-of-life; and examine which domains of health-related quality-of-life are associated with HIV stigma. Our analyses included people living with HIV not on treatment or in care, and the findings provide crucial evidence of the need for interventions to improve the wellbeing of people living with HIV.

## Methods

### Study design

Data were from the HPTN 071 (PopART) study, a three-arm, matched cluster-randomised controlled trial, implemented across 21 urban and peri-urban communities in Zambia (12 communities) and South Africa (9 communities) between 2013 and 2018. Each community was the catchment population for a government clinic, and in total, the study communities had a population of approximately 1 million people. The study measured the effect of a combination prevention package on HIV incidence. Arm A received the full intervention of door-to-door HIV testing plus access to HIV treatment for all PLHIV, Arm B received the intervention but followed national treatment guidelines (universal ART from 2016) and Arm C received standard care. Further details on the trial are available in the supplement (section S1), the trial protocol (https://www.hptn.org/research/studies/hptn071), and are described by Hayes et al.^21^

At baseline of the trial, a random sample of households were selected and visited in each community. All adults aged between 18 and 44 years in each household were identified and one selected at random. The selected adults were surveyed by trained research assistants in the respondent’s preferred language using electronic tablets and followed up annually for three years. This analysis used data from the final survey, conducted 36 months after the trial started, between 8th September 2017 and 7th July 2018.

The EuroQoL five dimensions, five levels questionnaire (EQ-5D-5L) was included in the survey to collect health-related quality-of-life data^20,22^. To complete the EQ-5D-5L, participants reported if they had problems, on a five-level scale (no problems, slight problems, moderate problems, severe problems or extreme problems/unable to do), in five domains: mobility (ability to walk around), self-care (ability to wash and dress), daily activities (ability to carry out their usual daily activities), pain, and anxiety/depression^22^. In South Africa the certified translation of the EQ-5D-5L was used, whereas in Zambia, members of the study team translated the questionnaire into Zambian dialects because no certified translation was available. Visuals with faces were shown alongside the written words describing each of the five levels to improve understanding of the levels of each domain. Each respondent was asked to indicate their health state against the most appropriate level of problems in each of the 5 dimensions, and the answer was recorded by the research assistant. The EQ-5D-5L has been widely used in the general population and with people living with HIV in high-income countries and low- and middle-income countries (LMICs)^19,20,23^. It has been shown to be reliable and valid in diverse settings^19,23^.

Participants who self-reported being HIV-positive were asked 11 questions about their experience of HIV stigma. These questions captured four composite stigma outcomes: internalised stigma (three questions), stigma experienced in the community in the last year (five questions), stigma experienced in healthcare settings in the last year (three questions) and any stigma experienced (11 questions), as described in section S1 and by Stangl et al.^24^ Internalised stigma occurs when people living with HIV apply negative feelings and beliefs associated with HIV to themselves^24^. Wording of the stigma measures was informed by conceptual frameworks^6^.

HIV status was assessed by venous blood testing. Detail on HIV testing is available in section S1.

### Statistical analysis

The study population was restricted to participants who self-reported living with HIV, which was confirmed by laboratory testing. Participants with missing data on health-related quality-of-life, stigma or other variables of interest were excluded, but participants did not need to be on treatment or in HIV care (section S2).

Firstly, a binary variable was created to summarise whether participants agreed with feeling any of three manifestations of internalised stigma. Two further binary variables captured whether participants had experienced stigma in a community setting and whether participants had experienced stigma in a healthcare setting. A fourth binary variable was also generated to encapsulate whether participants had experienced any stigma.

We then generated health-related quality-of-life profiles, which described the participants’ responses for each EQ-5D-5L domain^22^. Profiles were generated for two groups: people living with HIV who had never experienced HIV stigma, and people living with HIV who had experienced HIV stigma at least once. Each level of problems was given a quantitative score from one (no problems) to five (unable to/extreme problems), and Wilcoxon rank sum tests were applied to the scores to examine differences in each health-related quality-of-life domain between people living with HIV who had experienced stigma and people living with HIV who had not.

Participants’ responses to the EQ-5D-5L were collapsed into a binary health-related quality-of-life outcome, as suggested in the EQ-5D-5L User Guide, which captured whether they reported problems in any health-related quality-of-life domain^22^. If participants had a quantitative health-related quality-of-life score above one out of five for any of the domains, then they were considered to have problems in a health-related quality-of-life domain.

Logistic regression was used to assess the unadjusted and adjusted association between experiencing any HIV stigma and reporting problems in at least one health-related quality-of-life domain. Potential confounders included age, sex, education, wealth, religion, recreational drug use, tuberculosis status, and marital status. Cluster robust standard errors were computed to account for clustering by community. Wealth was assessed using an index generated from principal components analysis of data on assets owned by participants^6^.

After the variable capturing whether any HIV stigma had been experienced was assessed, the associations between the different types of stigma (community, healthcare setting, and internalised) and reporting problems in at least one health-related quality-of-life domain were examined. Three separate unadjusted models were developed, followed by a single adjusted model. The adjusted model included the three stigma variables and the potential confounders listed above, with cluster robust standard errors.

Finally, we conducted separate unadjusted univariable and separate adjusted logistic regression analyses for each of the health-related quality-of-life domains (mobility, self-care, daily activities, pain, and anxiety/depression) to determine which domains were associated with experiencing any HIV stigma. For each regression, the variable capturing whether any stigma had been experienced was the independent variable of interest and the health-related quality-of-life domain was the dependent variable. Adjusted models controlled for the potential confounders listed above and cluster robust standard errors were used.

All logistic regressions were complete case analyses. Calculated odds are in section S3.

Analyses were performed using R (version 4.0.3) and the package Miceadds^25,26^.

### Patient and public involvement

The public were involved through-out the HPTN 071 (PopART) research process. Before the trial began, individuals from existing representative structures, including members of community advisory boards from previous studies in the area, local opinion leaders, and government stakeholders, were consulted about study design^27^. A broad-brush survey approach was also used to provide a rapid assessment of the HIV prevention, treatment and care landscapes prior to trial initiation^28^. During the trial, multiple involvement and engagement mechanisms were employed to understand and improve the study, including meetings with community advisory boards for adults and adolescents, and connections with civil society groups^27^. Links with the public were also drawn upon at the end of intervention delivery to understand how results should be disseminated, with the first dissemination round using a community dialogue approach, which focused on what results meant to the communities^29^. Further dissemination of study results, such as those presented here, will continue to involve communities in decision making.

### Role of the funding source

The funders of the study had no role in the study design, data collection, data analysis, data interpretation, or writing of the report.

### Ethics

Ethical approval for the HPTN 071 (PopART) trial was obtained from institutional review boards at London School of Hygiene and Tropical Medicine (LSHTM), the University of Zambia, and Stellenbosch University. Participants provided written informed consent. The study was performed in accordance with relevant guidelines and regulations, including the Declaration of Helsinki.

## Results

There were 6,261 participants with laboratory confirmed HIV-positive status, 4,413 of whom self-reported living with HIV. After excluding 422 participants due to missing data on stigma, health-related quality-of-life or other variables, the study sample included 3991 self-reported people living with HIV (section S2).

There were more women (88%) than men (12%) in the sample (Table 1). Nearly half of participants (46%) were aged between 30 and 39.

**Table 1:** Summary statistics for 3991 people living with laboratory-confirmed HIV in Zambia and South Africa who responded to questions measuring their experience of stigma in the fourth survey of the HPTN 071 (PopART) cohort, September 2017 to July 2018.

Variable	Variable categories	N (%) of estimation sample
Health-related quality-of-life	No problems reported in any domain	3517 (88%)
	Problems reported in at least one domain	474 (12%)
Enacted community stigma	Never experienced enacted stigma in a community setting	3298 (83%)
	Experienced stigma in a community setting at least once	693 (17%)
Enacted healthcare setting stigma	Never experienced stigma in a healthcare setting	3833 (96%)
	Experienced stigma in a healthcare setting at least once	158 (4%)
Internalised stigma	Do not have internalised stigma	3439 (86%)
	Have internalised stigma	552 (14%)
Sex	Female	3525 (88%)
	Male	466 (12%)
Age	18–24 years	308 (8%)
	25–29 years	616 (15%)
	30–34 years	849 (21%)
	35–39 years	995 (25%)
	40–44 years	895 (22%)
	> 45 years	328 (8%)
Education level	Primary school education or less (< grade 8)	1101 (28%)
	Secondary school education (grade 8–12)	2715 (68%)
	Further/higher education	175 (4%)
Wealth index^a^	Wealth quintile 1 (poorest)	958 (24%)
	Wealth quintile 2	812 (20%)
	Wealth quintile 3	921 (23%)
	Wealth quintile 4	759 (19%)
	Wealth quintile 5 (richest)	541 (14%)
HIV care status	Not in HIV care	336 (8%)
	In HIV care, not on antiretroviral therapy	240 (6%)
	In HIV care, on antiretroviral therapy	3415 (86%)
Marital status	Not married	1926 (48%)
	Married	2065 (52%)
Religion	Christian	3733 (94%)
	Other	258 (6%)
Recreational drug use	Has not used recreational drugs in the last 12 months	3911 (98%)
	Has used recreational drugs in the last 12 months	80 (2%)
Tuberculosis status	Have not been told they had tuberculosis in the last 12 months	3894 (98%)
	Have been told they had tuberculosis in the last 12 months	97 (2%)
Country	Zambia	2637 (66%)
	South Africa	1354 (34%)

^a^Wealth was assessed using an asset index generated from principal components analysis of data on assets owned by an individual.

Problems in at least one health-related quality-of-life domain were reported by 515 participants (12%), and 1,034 participants (35%) reported experiencing HIV stigma at least once. The Wilcoxon tests, which analysed problem severity coded as a score from one to five, showed that, for all health-related quality-of-life domains, there was a significant difference in the proportion of people reporting each level of problem severity between people living with HIV who had and had not experienced HIV stigma (Table 2). This unadjusted analysis suggested that having experienced stigma was associated with poorer health-related quality-of-life in all five domains.

**Table 2:** Degree of problems in five health-related quality-of-life domains by HIV stigma experience for 3991 people living with HIV from 21 study communities in South Africa and Zambia.

Health-related quality-of-life domain	Never experienced any stigma (n = 2957)	Experienced stigma at least once (n = 1034)	*p* value^a^
Mobility			
No problems	2913 (99%)	995 (96%)	
Slight problems	35 (1%)	32 (3%)	
Moderate problems	5 (< 1%)	5 (< 1%)	
Severe problems	3 (< 1%)	2 (< 1%)	
Unable to walk about	1 (< 1%)	0 (0%)	*p* < 0.001
Self-care			
No problems	2924 (99%)	1012 (98%)	
Slight problems	24 (1%)	20 (2%)	
Moderate problems	4 (< 1%)	1 (< 1%)	
Severe problems	3 (< 1%)	0 (0%)	
Unable to wash or dress	2 (< 1%)	1 (< 1%)	*p* = 0.017
Usual activities			
No problems	2891 (98%)	990 (96%)	
Slight problems	53 (2%)	37 (4%)	
Moderate problems	11 (< 1%)	6 (1%)	
Severe problems	1 (< 1%)	1 (< 1%)	
Unable to do usual activities	1 (< 1%)	0 (0%)	*p* = 0.001
Pain/discomfort			
No pain/discomfort	2768 (94%)	909 (88%)	
Slight pain/discomfort	145 (5%)	98 (9%)	
Moderate pain/discomfort	32 (1%)	20 (2%)	
Severe pain/discomfort	10 (< 1%)	5 (< 1%)	
Extreme pain/discomfort	2 (< 1%)	2 (< 1%)	*p* < 0.001
Anxiety/depression			
Not anxious/depressed	2868 (97%)	945 (91%)	
Slightly anxious/depressed	65 (2%)	56 (5%)	
Moderately anxious/depressed	15 (1%)	22 (2%)	
Severely anxious/depressed	7 (< 1%)	9 (1%)	
Extremely anxious/depressed	2 (< 1%)	2 (< 1%)	*p* < 0.001

^a^P values are from Wilcoxon rank-sum tests for difference between those who never experienced HIV stigma and those who experienced HIV stigma at least once, with problem severity for each health-related quality-of-life domain coded as a score from one to five.

In univariable regression analyses exploring the association between experiencing any HIV stigma and participants’ health-related quality-of-life, the odds of reporting problems in at least one health-related quality-of-life domain were 2.10 (95% confidence intervals, CI 1.72–2.56, p < 0.001) times higher if a participant experienced HIV stigma at least once, compared to never experiencing HIV stigma (Table 3).This finding remained stable on adjusting for confounders and introducing cluster robust standard errors; participants who had experienced any HIV stigma had more than twice the odds of reporting problems in at least one health-related quality-of-life domain compared to those who had not (adjusted odds ratio, aOR 2.08, 95% CI 1.55–2.79, p < 0.001). Using recreational drugs in the last year (OR 1.94, 95% CI 1.00–3.75, *p* = 0.050), and being diagnosed with tuberculosis in the last year (OR 2.15, 95% CI 1.14–4.04, *p* = 0.018) were also associated with higher odds of reporting problems in at least one health-related quality-of-life domain.

**Table 3:** Univariable and multivariable analysis of the association between experiencing any form of stigma and having reported problems in at least on health-related quality-of-life domain among 3991 people living with HIV from 21 study communities in South Africa and Zambia.

Variable	Participants reporting problems in at least one health-related quality-of-life domain	Univariable logistic models		Multivariable logistic model^a^	
		Odds ratio (95% confidence interval)^b^	*P* value	Odds ratio (95% confidence interval)^b^	*P* value
Never experienced any stigma (base)	285/2957 (10%)	1	–	1	–
Experienced any stigma at least once	189/1034 (18%)	2.10 (1.72–2.56)	< 0.001	2.08 (1.55–2.79)	< 0.001
Female (base)	422/3525 (12%)	1	–	1	–
Male	52/466 (11%)	0.92 (0.67–1.24)	0.610	0.88 (0.72–1.07)	0.184
Age 18–24 y (base)	37/308 (12%)	1	–	1	–
Age 25–29 y	78/616 (13%)	1.06 (0.70–1.63)	0.778	1.11 (0.70–1.75)	0.646
Age 30–34 y	99/849 (12%)	0.97 (0.65–1.46)	0.869	1.03 (0.66–1.61)	0.895
Age 35–39 y	105/995 (11%)	0.86 (0.59–1.30)	0.473	0.96 (0.67–1.37)	0.812
Age 40–44 y	112/895 (13%)	1.05 (0.71–1.57)	0.818	1.12 (0.76–1.66)	0.559
Age 45 +	43/328 (13%)	1.11 (0.69–1.77)	0.677	1.23 (0.80–1.89)	0.339
Does not use recreational drugs (base)	457/3911 (12%)	1	–	1	–
Uses recreational drugs	17/80 (21%)	2.04 (1.15–3.43)	0.010	1.94 (1.00–3.75)	0.050
Not been told they have tuberculosis in the last year (base)	454/3894 (12%)	1	–	1	–
Been told they have tuberculosis in the last year	20/97 (21%)	1.97 (1.16–3.18)	0.008	2.15 (1.14–4.04)	0.018
Not married (base)	214/1926 (11%)	1	–	1	–
Married	233/2065 (11%)	0.89 (0.73–1.08)	0.230	0.89 (0.78–1.03)	0.109
Christian (base)	448/3733 (12%)	1	–	1	–
Other religion	26/258 (10%)	0.82 (0.53–1.22)	0.356	0.85 (0.57–1.26)	0.416
Primary school education (base)	138/1101 (13%)	1	–	1	–
Secondary school education	316/2715 (12%)	0.92 (0.74–1.14)	0.439	0.95 (0.68–1.34)	0.773
Further/higher education	20/155 (13%)	0.90 (0.53–1.45)	0.680	0.92 (0.55–1.53)	0.745
Wealth quintile 1, poorest (base)	110/958 (11%)	1	–	1	–
Wealth quintile 2	92/812 (11%)	0.99 (0.73–1.32)	0.920	1.05 (0.78–1.41)	0.747
Wealth quintile 3	99/921 (11%)	0.93 (0.70–1.24)	0.613	0.99 (0.73–1.35)	0.970
Wealth quintile 4	107/759 (14%)	1.27 (0.95–1.68)	0.106	1.33 (0.97–1.83)	0.077
Wealth quintile 5, richest	66/541 (12%)	1.07 (0.77–1.48)	0.679	1.12 (0.80–1.58)	0.502

^a^The multivariate model is adjusted for sex, age, recreational drug use, tuberculosis status, marital status, religion, education, and wealth, with cluster robust standard errors to account for clustering by community.

^b^An odds ratio greater than one shows those not in the base category are more likely to report problems in at least one health-related quality-of-life domain. For categorical variables, each category is compared with the base category.

When the three types of stigma were analysed, HIV stigma experienced in the community was the most frequently reported (17%), followed by internalised stigma (14%) and stigma experienced in healthcare settings (4%) (Table 1). In unadjusted analyses of the association between the three different types of stigma and health-related quality-of-life, participants who had experienced internalised stigma, stigma in the community, or stigma in a healthcare setting had higher odds of reporting problems in at least one health-related quality-of-life domain, with unadjusted ORs of 2.29 (95% CI 1.81–2.88, p < 0.001), 1.86 (95% CI 1.48–2.32, p < 0.001), and 1.56 (95% CI 1.00–2.35, *p* = 0.040), respectively (Table 4).

**Table 4:** The association between each of the three types of HIV stigma, and health-related quality-of-life among 3991 people living with HIV from 21 study communities in South Africa and Zambia.

Variable	Participants reporting problems in at least one health-related quality-of-life domain	Univariable logistic models		Adjusted logistic model^a^	
		Odds ratio (95% confidence interval)^b^	*P* value	Odds ratio (95% confidence interval)^b^	*P* value
Never experienced community stigma (base)	349/3298 (11%)	1	–	1	–
Experienced community stigma at least once	125/693 (18%)	1.86 (1.48–2.32)	< 0.001	1.51 (1.16–1.98)	0.002
Never experienced stigma in a healthcare setting (base)	447/3833 (12%)	1	–	1	–
Experienced stigma in a healthcare setting at least once	27/158 (17%)	1.56 (1.00–2.35)	0.040	1.04 (0.68–1.58)	0.850
Have not experienced internalised stigma (base)	358/3439 (10%)	1	–	1	–
Have experienced internalised stigma	116/552 (21%)	2.29 (1.81–2.88)	< 0.001	1.98 (1.54–2.54)	< 0.001

^a^The adjusted model includes community stigma, healthcare stigma, internalised stigma, sex, age, recreational drug use, tuberculosis status, marital status, religion, education, and wealth, with cluster robust standard errors to account for clustering by community.

^b^An odds ratio greater than one indicates those not in the base category are more likely to report problems in at least one health-related quality-of-life domain.

After adjusting for potential confounders and using cluster robust standard errors, the odds of reporting problems in at least one health-related quality-of-life domain were nearly twice as high in those who had experienced internalised stigma compared to those who had not (aOR 1.98, 95% CI 1.54–2.54, p < 0.001). The odds of reporting problems in at least one health-related quality-of-life domain were 51% higher for people living with HIV who had experienced community stigma at least once compared to those who had not (aOR 1.51, 95% CI 1.16–1.98, *p* = 0.002). There was no association between experiencing healthcare setting stigma and reporting problems in at least one health-related quality-of-life domain after adjustment (aOR 1.04, 95% CI 0.68–1.58, *p* = 0.850) (Table 4).

**Figure 1:**
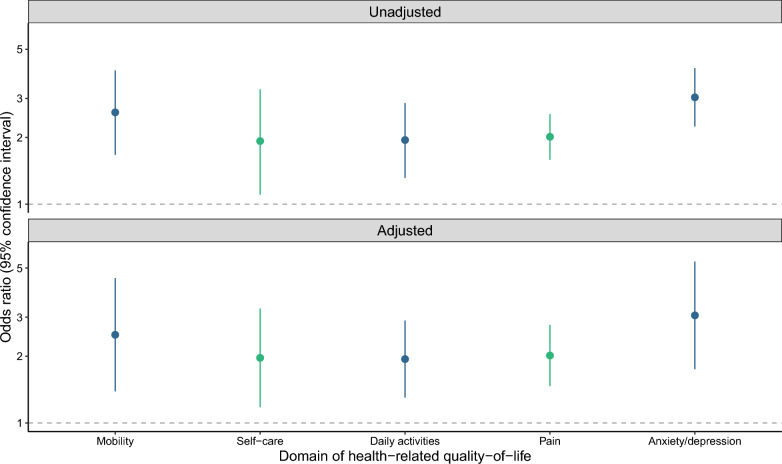
The association between experiencing any HIV stigma and reporting problems in five dimensions of health-related quality-of-life among 3991 people living with HIV from 21 study communities in South Africa and Zambia. Not experiencing any HIV stigma is the base category. An odds ratio greater than one shows those not in the base category are more likely to report “problems” in that health-related quality-of-life domain. Adjusted models include the covariates age, sex, education, wealth index, religion, recreational drug use, tuberculosis status, and marital status, with cluster robust standard errors to account for clustering by community.

Unadjusted and adjusted regression analyses of the associations between experiencing any stigma and health-related quality-of-life domains showed that experiencing any HIV stigma was associated with increased odds of reporting problems in all health-related quality-of-life domains (Fig. 1). In unadjusted analyses, participants who experienced any type of stigma had higher odds of reporting problems with mobility (OR 2.59, 95% CI 1.67–4.02, p < 0.001), self-care (OR 1.93, 95% CI 1.10–3.30, *p* = 0.018), performing daily activities (OR 1.95, 95% CI 1.31–2.86, *p* = 0.001), pain (OR 2.01, 95% CI 1.58–2.55, p < 0.001), and anxiety/depression (OR 3.03, 95% CI 2.24–4.11, p < 0.001) than those who had never experienced HIV stigma. In adjusted analyses, results were similar. The odds of reporting problems among those who had experienced HIV stigma were 2.5 (OR 2.50, 95% CI 1.39–4.50, *p* = 0.002) times higher for mobility, 97% (OR 1.97, 95% CI 1.18–3.29, *p* = 0.010) higher for self-care, 94% (OR 1.94, 95% CI 1.30–2.90, *p* = 0.001) higher for performing daily activities, 2.01 (OR 2.01, 95% CI 1.47–2.77, p < 0.001) times higher for pain, and 3.06 (OR 3.06, 95% CI 1.75–5.35, p < 0.001) times higher for anxiety/depression.

## Discussion

This study analysed the association between HIV stigma and the health-related quality-of-life of people who self-reported living with HIV, in 21 urban and peri-urban communities across South Africa and Zambia. 17% of participants reported experiencing HIV stigma in the community, 4% had experienced stigma in healthcare settings, and 14% of participants had internalised stigma. Experiencing internalised stigma or stigma in the community was associated with twice, and one and a half times higher odds, respectively, of reporting worse health-related quality-of-life outcomes. We also found that problems in all domains of health-related quality-of-life contributed to the association between HIV stigma and reduced health-related quality-of-life. Experiencing stigma in a healthcare setting was not associated with reporting worse health-related quality-of-life.

Our findings build on previous studies linking internalised stigma with lower health-related quality-of-life among people living with HIV who knew their status^4,16^. Moreover, several studies have found that people living with HIV with internalised stigma have an increased likelihood of developing depression, and our analysis found those who experienced any stigma had three times higher odds of reporting problems with anxiety or depression^30^. Likewise, community stigma has previously been linked to avoidance of healthcare, and poor mental health, supporting our finding that experiencing community stigma is negatively associated with health-related quality-of-life among people living with HIV^31^. Our stigma prevalence values differ slightly from those in previous reports relating to the HPTN 071 (PopART) trial due to the different study sample selected here^6^.

Our finding that experiencing any HIV stigma was associated with increased odds of reporting problems in all the health-related quality-of-life domains aligns with some, but not all previous research. Specifically, our finding mirrors the results of a study in Nigeria, but differs from results of two studies in Ethiopia, wherein associations were only found for some health-related quality-of-life domains^5,8,17^. The difference between the results of the Ethiopian studies and our study may be due to diversity in participant characteristics, such as age, lifestyle, and perception of personal beliefs^5^. Nevertheless, it should also be recognised that the differences may be a consequence of the use of various health-related quality-of-life and stigma measures, which capture distinct concepts and so may reveal differing associations.

An unexpected finding of our study was that stigma experienced in a healthcare setting was not associated with worse health-related quality-of-life in adjusted analyses. Qualitative stigma data from the HPTN 071 (PopART) trial consistently and strongly indicates concerns about people living with HIV being seen accessing HIV services in healthcare settings, and the role of group identity, spatial layout, client flow, and items that signify HIV, in driving healthcare setting stigma^32^. Thus, the qualitative data does not support this finding. In our study, healthcare setting stigma was the least frequently experienced stigma type, consequently the study may have been underpowered to detect significant effects. Additionally, the EQ-5D-5L is a generic measure of health, so might not be sufficiently sensitive to identify indirect adverse impacts of healthcare stigma, such as altered healthcare-seeking behaviours^9^. The HPTN 071 (PopART) trial was also associated with the healthcare system, which may have led to under-reporting of stigma experienced in healthcare settings. Lastly, the three items used to assess healthcare setting stigma may not have captured less overt stigma, which may be more prevalent in healthcare settings in sub-Saharan Africa, given the long history of stigma reduction efforts^33^. Nonetheless, our finding is potentially important because most current literature on interventions to reduce HIV stigma focuses on healthcare settings^34^. Stigma occurs in many places, with six main settings identified by UNAIDS: the healthcare sector, the education sector, the workplace, the justice system, families and communities, and emergency and humanitarian settings^35^. Our findings provide crucial guidance to policymakers in South Africa and Zambia to refocus stigma reduction policies beyond the healthcare sector.

The main strength of this study is the substantial sample of people living with HIV. Our study is among the largest and most robust analyses of associations between HIV stigma and health-related quality-of-life in Africa. We included nearly 4,000 people living with HIV from 21 communities in two countries. Moreover, while previous studies have often evaluated health-related quality-of-life in people living with HIV who were enrolling into clinical care or on treatment, our data were from people living with HIV in the community^4,5,8,16,17^. Approximately one in ten of the included people living with HIV were not in care^20^. This gives a more representative insight into the relationship between stigma and health-related quality-of-life. Additionally, our measures of the core manifestations of HIV stigma and of health-related quality-of-life had been validated previously and self-reported HIV-positive status was confirmed via laboratory testing. Finally, the scope of the PopART trial allowed many confounders, selected based on research in similar populations, to be adjusted for.

Our study had limitations. Males were underrepresented, possibly due to selection bias; the survey was administered during the day, when men may have been more likely to be at work. There are also fewer men than women living with HIV in South Africa and Zambia. This limits the generalisability of our findings to men, particularly as some studies have found gender differences in stigma and health-related quality-of-life^6,17^. Additionally, we excluded people living with HIV who did not self-report living with HIV, as they were not asked about HIV stigma. This excluded people living with HIV who did not know their status or chose not to report their status. Excluding people who chose not to report their status may result in sample selection bias, because individuals who have experienced stigma may be less likely to disclose their status, perhaps leading to an underestimation of the prevalence of HIV stigma and its associations with health-related quality-of-life. Moreover, despite CD4 cell count (an indication of immune system health) being identified as a potential confounder, we were unable to adjust for this as data on CD4 cell counts were not collected^19^. Finally, this study focused on experienced and internalised stigma, but did not investigate anticipated stigma. Therefore, future work should explore the role of anticipated stigma. Future work could also examine the impact of the HPTN 071 (PopART) combination prevention intervention on the relationship between stigma and health-related quality-of-life. While previous research has demonstrated that the intervention had no effect on overall stigma or overall health-related quality-of-life, the intervention may have modified the effect of stigma on health-related quality-of-life and this could be explored to complement our findings^36,37^.

Over recent years, studies have shown that progress has been made in the design of interventions addressing both stigma experienced in the community and internalised stigma^33^. Interventions to reduce community stigma are often information-based, aiming to educate people living with HIV and the wider community about HIV to improve attitudes^35^. There is existing evidence that these approaches tend to result in some reduction in stigmatising behaviour, however, studies could not always demonstrate quantifiably measurable change^38^. Evidence suggests that interventions combining broad information-based campaigns with strategies targeting community leaders and groups who are influential in setting norms and practices may be more effective^9^. Furthermore, internalised stigma has been successfully addressed through interventions including formal psychological support, treatment buddies, and support groups^9^. Studies have shown that substantial challenges exist in scaling interventions for internalised stigma, especially in LMICs, but this is important as internalised stigma has been linked with poorer viral suppression^39,40^. Implementing and evaluating evidence-based interventions to reduce both internalised stigma and stigma experienced in the community should be a priority.

Our analysis demonstrates the use of the EQ-5D-5L, a generic measure of health-related quality-of-life, to detect adverse effects of stigma. In future, this instrument could be employed to assess the benefits of stigma interventions and compare them against a wide range of other health interventions that are assessed with generic measures^41^. For example, the benefit estimates could be used in comparative cost-effectiveness analyses of stigma interventions, to determine the societal value of such interventions against other health interventions^19,20^. Eventually, these estimates may be used in comparative cost-effectiveness league tables, such as those used to determine health packages for universal health coverage^41^. This provides invaluable guidance to policymakers navigating constrained health budgets and may inform decisions on funding allocations that stigma interventions receive^19,20^.

## Conclusions

Our study showed that internalised stigma and stigma experienced in the community were associated with worse health-related quality-of-life, but stigma experienced in healthcare settings was not associated with health-related quality-of-life. These findings indicate that there is a need for research into, and implementation of, cost-effective stigma reduction interventions that focus on internalised stigma and community stigma, in South Africa and Zambia. Efforts to reduce stigma must continue at multiple levels to improve the lives of people living with HIV and achieve the new societal enabler targets for HIV, which are key to reaching global HIV goals by 2030.

### Supplementary Information

Supplementary Information.

## Data Availability

The data archive is held at Fred Hutchinson Cancer Center, Seattle, WA, USA. Requests can be sent to HPTN‐Data‐Access@scharp.org.
