# Supplementary material for: Associations between HIV stigma and health-related quality-of-life among people living with HIV: cross-sectional analysis of data from HPTN 071 (PopART)
Source: Sci Rep. 2024 Jun 4;14:12835. doi: 10.1038/s41598-024-63216-3 (PMC11150264; doi:10.1038/s41598-024-63216-3)
Supplement: Supplementary file 1 — Supplementary Information. [file 41598_2024_63216_MOESM1_ESM.pdf]

Supplementary Information for Associations between HIV stigma and health-related quality-of-life among people living with HIV: Cross-sectional analysis of data from HPTN 071 (PopART).

Authors: Emily Hall, Katherine Davis, Julius Ohrnberger, Michael Pickles, Simon Gregson, Ranjeeta Thomas, James R Hargreaves, Triantafyllos Pliakas, Justin Bwalya, Rory Dunbar, Tila Mainga, Kwame Shanaube, Graeme Hoddinott, Virginia Bond, Peter Bock, Helen Ayles, Anne L Stangl, Deborah Donnell, Richard Hayes, Sarah Fidler, Katharina Hauck, and the HPTN 071 (PopART) study team.

Corresponding author: Katherine Davis, MRC Centre for Global Infectious Disease Analysis and the Abdul Latif Jameel Institute for Disease and Emergency Analytics, School of Public Health, Imperial College London, London, UK. Email: k.davis18@imperial.ac.uk

<u>Contents</u>

Section 1 – Supplementary information on HPTN 071 (PopART) study 2

Section 2 – Supplementary Figures and Tables 4

Section 3 – Calculated Odds 5

References 6

Section 1: Supplementary information on HPTN 071 (PopART) study

S1a: Trial design

The HPTN 071 (PopART) trial was completed between 2013 and 2018, in 21 large, urban communities in Zambia (n=12) and South Africa (n=9). In total, the communities had a population of approximately 1 million people and each community was the catchment population for a government clinic.

At the start of the trial, the communities were split into seven triplets, matched on location and estimated HIV prevalence. The three communities in each triplet were then randomly assigned to three trial arms (two intervention arms and a control arm), using restricted randomisation to ensure balance across the trial arms with respect to population size, baseline ART coverage (the percentage of people living with HIV who were receiving ART), and HIV prevalence. Arm A communities received a combination prevention intervention with universal ART. Arm B communities received a combination prevention intervention with ART provided according to local guidelines. Arm C communities did not receive a combination prevention intervention but received standard care at government clinics, including HIV testing and ART offered according to local guidelines. The combination prevention intervention included, amongst other components, home-based HIV counselling and testing delivered by community HIV care provider teams, who also supported linkage to HIV care, encouraged ART adherence, provided condoms, and promoted a package of prevention strategies among HIV-negative individuals, including voluntary medical male circumcision. In arms B and C, the clinics initially provided ART at a CD4 threshold of 350 cells per microliter, however this increased to 500 cells per microliter in 2014 and universal ART was offered from 2016. After the introduction of universal ART, the two intervention arms were equivalent.

The effect of the intervention was measured in a population cohort (enrolled from December 2013 to March 2015) that included one randomly selected adult aged 18 to 44 years from a random sample of households in each community. Participants were surveyed at baseline and after 12, 24, and 36 months. The original enrolment target (2500 adults per community) was not reached at baseline, so additional participants were enrolled at 12 months and at 24 months, excluding households sampled previously.

At each survey, participants were interviewed by a field research assistant using a structured questionnaire that included the collection of demographic, socioeconomic, and behavioural data as well as data related to HIV and health-related quality-of-life. After the interview, blood was collected by a research nurse, who also offered rapid HIV testing to all participants.

The analysis presented here used cross-sectional data from the final survey, conducted 36 months after the start of the HPTN 071 (PopART) trial, between 8th September 2017 and 7th July 2018.

The sample size for the HPTN 071 (PopART) trial determined the sample size used in this analysis. Before the trial began, the sample size for HPTN 071 (PopART) was determined from initial projections of the intervention effect on HIV incidence from mathematical modelling, which indicated that around 2,500 participants were required per community.

S1b: Blood sample and HIV testing

Blood samples were analysed in-country using a single 4th generation assay (Architect HIV Ag/Ab Combo Assay, Abbott Diagnostics, Delkenheim Germany). Further testing was performed at the HIV Prevention Trials Network (HPTN) Laboratory Center (Baltimore, MD, USA). Samples that had reactive results in-country were tested with a second 4th generation assay (GS HIV Combo Assay, Bio-Rad Laboratories, Redmond, Wa). For quality assurance, 10% of the samples that had non-reactive results in-country were tested again using the Architect HIV Ag/Ab Combo Assay. Samples with discrepant/discordant test results were tested with additional assays to determine HIV status.

S1c: Stigma composite variables

The stigma composite variables were derived from 11 questions that people living with HIV were asked.

Table S1 Questions capturing the composite stigma outcomes

**Composite stigma variable**	**Questions asked in the creation of composite variable**	**Four-point Likert scale response options**
Internalised stigma	*Three questions:*<br>Please tell me how strongly you agree or disagree with the following statements:<br>• I have lost respect or standing in the community because of my HIV status.<br>• I think less of myself because of my HIV status.<br>• I have felt ashamed because of my HIV status.	strongly agree; agree; disagree; strongly disagree
Stigma experienced in the community	*Five questions:*<br>Please tell us how often the following things have happened to you, or whether you think they have happened to you, because of your HIV status in the last 12 months:<br>• People have talked badly about me because of my HIV status.<br>• I have been verbally insulted, harassed and/or threatened because of my HIV status.<br>• I have been physically assaulted because of my HIV status.<br>• I have felt that people have not wanted to sit next to me because of my HIV status.<br>• Someone else disclosed my HIV status without my permission.	never; once; a few times; often
Stigma experienced in a healthcare setting	*Three questions:*<br>Please tell us how often the following things have happened to you, or whether you think they have happened to you, because of your HIV status in the last 12 months:<br>• Healthcare workers talked badly about me because of my HIV status.<br>• A health worker disclosed my HIV status without my permission,<br>• I have been denied health services because of my HIV status.	never; once; a few times; often
Any stigma experienced	*All 11 questions from internalised stigma, stigma experienced in the community and stigma experienced in a healthcare setting.*	..

Section 2: Supplementary Tables and Figures

Fig. S1 Flowchart for analysis sample

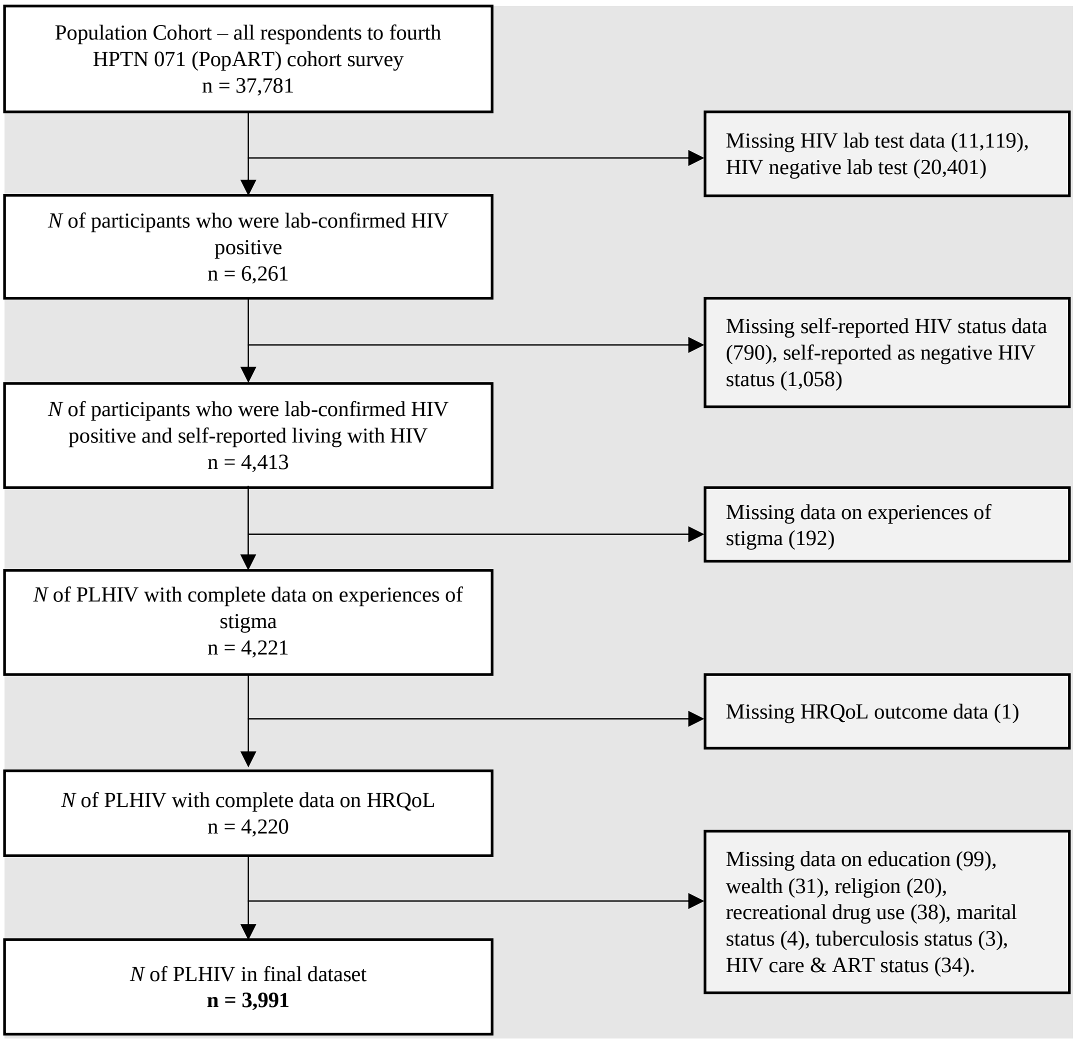

HPTN: HIV Prevention Trials Network, PLHIV: people living with HIV, HRQoL: health-related quality-of-life, ART: antiretroviral therapy

Section 3: Calculated Odds

The association between three types of HIV stigma, and health-related quality-of-life among 3,991 people living with HIV from 21 study communities in South Africa and Zambia

For a woman living with HIV, aged 35-39 from the Dambwe community, who had reached secondary level of education, was in the poorest wealth quintile, was Christian, married, and had not taken recreational drugs in the last year, nor been told she has tuberculosis in the last year and…

> Had ***not*** experienced HIV stigma, the odds of reporting problems in at least one domain of HRQoL was 0.08.
>
> Had experienced HIV stigma in ***healthcare*** settings at least once, the odds of reporting problems in at least one domain of HRQoL was 0.09.
>
> Had experienced HIV stigma in the ***community*** at least once, the odds of reporting problems in at least one domain of HRQoL was 0.13.
>
> Had experienced ***internalised*** stigma, the odds of reporting problems in at least one domain of HRQoL was 0.17.

The association between experiencing any HIV stigma and reporting problems in five dimensions of health-related quality-of-life among 3,991 people living with HIV from 21 study communities in South Africa and Zambia

For a woman living with HIV aged 35-39 from the Dambwe community, who reached secondary level of education, was in the poorest wealth quintile, was Christian, married, and had not taken recreational drugs in the last year, nor been told she had tuberculosis in the last year…

> The odds of reporting problems in the domain of ***mobility*** were 0.008 for those who had not experienced HIV stigma, and 0.022 for those who had experienced HIV stigma at least once.
>
> The odds of reporting problems in the domain of ***self-care*** were 0.004 for those who had not experienced HIV stigma, and 0.007 for those who had experienced HIV stigma at least once.
>
> The odds of reporting problems in the domain of ***daily activities*** were 0.035 for those who had not experienced HIV stigma, and 0.047 for those who had experienced HIV stigma at least once.
>
> The odds of reporting problems in the domain of ***pain*** were 0.047 for those who had not experienced HIV stigma, and 0.096 for those who had experienced HIV stigma at least once.
>
> The odds of reporting problems in the domain of ***anxiety/depression*** were 0.033 for those who had not experienced HIV stigma, and 0.100 for those who had experienced HIV stigma at least once.

References:

Thomas R, Burger R, Harper A, et al. Differences in health-related quality of life between HIV-positive and HIV-negative people in Zambia and South Africa: a cross-sectional baseline survey of the HPTN 071 (PopART) trial. Lancet Glob Heal 2017;5:e1133–41. https://doi.org/10.1016/S2214-109X(17)30367-4

Hargreaves JR, Krishnaratne S, Mathema H, et al. Individual and community-level risk factors for HIV stigma in 21 Zambian and South African communities: Analysis of data from the HPTN071 (PopART) study. AIDS 2018;32:783–93. https://doi.org/10.1097/QAD.0000000000001757

Hayes RJ, Ayles H, Beyers N, et al. HPTN 071 (PopART): Rationale and design of a cluster-randomised trial of the population impact of an HIV combination prevention intervention including universal testing and treatment - a study protocol for a cluster randomised trial. Trials 2014;15:1–17. https://doi.org/10.1186/1745-6215-15-57

Hargreaves JR, Stangl A, Bond V, et al. HIV-related stigma and universal testing and treatment for HIV prevention and care: design of an implementation science evaluation nested in the HPTN 071 (PopART) cluster-randomized trial in Zambia and South Africa. Health Policy Plan 2016;31:1342–54. https://doi.org/10.1093/heapol/czw071
